# Medical Students’ Experiences With an Integrated Surgical Suturing Training Module Using Simulation Models and Asynchronous Videos: Interpretive Qualitative Study

**DOI:** 10.2196/90563

**Published:** 2026-07-09

**Authors:** Álvaro Herrera Alcaíno, Consuelo Vade Martínez, Benjamín Painemal Rivera, Daniela Serey Torres, Sebastián Puentes Bravo, Ignacio Leal Lizama, Josefina Darrigol Parra, Camila Riquelme Bahamondes

**Affiliations:** 1 Faculty of Medicine, Universidad San Sebastián, Lota 2465, Santiago, Metropolitan Region, Chile, 56 966696925; 2 Universidad San Sebastián, Santiago, Metropolitan Region, Chile; 3 Faculty of Medicine, Universidad San Sebastián, Concepción, Biobio Region, Chile

**Keywords:** medical education, surgical education, simulation-based learning, suturing, qualitative research

## Abstract

**Background:**

Digital educational resources, including asynchronous video-based materials and simulation models, are increasingly used in undergraduate medical education to support procedural skills training. Although both approaches have demonstrated educational value, there is limited qualitative evidence on how the pedagogical integration of these approaches is experienced in real-world clinical training environments, particularly during time-constrained surgical internships.

**Objective:**

This study aimed to explore medical students’ and an instructor’s experiences with an integrated suturing skills module combining asynchronous instructional videos and simulation-based practice during a surgical internship.

**Methods:**

An interpretive qualitative study was conducted with sixth-year medical students during a surgical internship at a medical school in Chile. The intervention consisted of a suturing simulation module supported by asynchronous instructional videos. Data were collected through 2 focus groups with 20 students and a semistructured interview with 1 instructor after completion of the module. All sessions were audio-recorded, transcribed verbatim, and pseudonymized. Data were analyzed using reflexive thematic analysis following an inductive approach. The Technological Pedagogical Content Knowledge framework was used as a sensitizing lens during the interpretive phase. Ethics approval was obtained prior to data collection, and all participants provided informed consent.

**Results:**

Participants described asynchronous videos as facilitating prior preparation by allowing flexible, self-paced review of procedural steps, which supported students’ perceived readiness for hands-on practice. The integration of video-based resources with simulation-based training was perceived as enabling more focused engagement during supervised sessions, shifting attention from procedural recall to technical execution. Participants also reported perceived increases in confidence when approaching early clinical tasks. However, logistical constraints, including limited time for individualized feedback and challenges related to the physical learning environment, were identified. Some participants also expressed ongoing uncertainty regarding specific techniques despite prior video exposure.

**Conclusions:**

The integration of asynchronous instructional videos with simulation-based practice was perceived as a coherent educational strategy that supported students’ preparation for procedural training within a demanding clinical context. The findings highlight the potential of asynchronous resources to function as preparatory scaffolding that may enable more efficient use of supervised training time. This study contributes to the qualitative understanding of how integrated digital and simulation-based approaches are experienced in surgical internships, supporting their consideration in curriculum design while emphasizing the need for careful implementation and contextual adaptation.

## Introduction

Digital educational resources, including asynchronous video-based learning materials and simulation models, have been increasingly incorporated into undergraduate medical education to support the acquisition of procedural skills [1-3]. In surgical education, these approaches have been used to facilitate deliberate practice, promote learner autonomy, and enhance preparedness for hands-on clinical training, particularly in settings where opportunities for supervised practice may be limited [2-6]. Recent studies have further highlighted the accelerated adoption of digital and blended learning strategies in health professions education, particularly following shifts toward flexible and technology-enhanced learning environments [7,8].

Instructional videos have been widely adopted as preparatory tools for procedural training, allowing learners to review techniques repeatedly, control the pace of learning, and access content flexibly according to their individual needs [6,9]. Prior research has shown that video-based resources can support self-regulated learning and procedural familiarization, which may be especially relevant during clinically intensive training periods such as surgical clerkships or internships [10,11]. However, most studies have focused on the effectiveness or usability of videos as isolated educational tools, with less attention being paid to how they are integrated into broader pedagogical designs and authentic clinical training environments [3,5,11]. More recent literature suggests that the educational impact of video-based learning depends not only on access to content but also on how these resources are pedagogically integrated into structured learning activities [12].

Simulation-based training has also been recognized as a key strategy for teaching surgical skills in a safe and controlled environment, enabling repeated practice without risk to patients [12,13]. Simulation models have been shown to support the development of technical skills and complement clinical exposure, particularly for basic surgical procedures such as suturing [13-15]. In Latin American medical education contexts, simulation-based approaches for teaching surgical skills have likewise been described as relevant and feasible, highlighting their educational value in settings characterized by high clinical demands and limited protected training time [1,14]. Recent work has also emphasized the role of simulation in supporting early skill acquisition and confidence in resource-constrained and clinically intensive training environments [15].

Despite the growing use of both asynchronous videos and simulation in surgical education, there is limited qualitative evidence exploring how students and instructors experience the combined and pedagogically integrated use of these resources within authentic clinical training contexts. In particular, little is known about how such integrated approaches are perceived and used by students during surgical internships, where time constraints, workload, and clinical responsibilities may shape engagement with educational interventions.

Understanding learners’ and instructors’ experiences with integrated digital and simulation-based strategies may provide valuable insights into how these approaches function in practice and how they can be meaningfully aligned with pedagogical goals and clinical training demands.

The aim of this study was to explore medical students’ and an instructor’s experiences with an integrated suturing skills module combining asynchronous instructional videos and simulation-based practice during a surgical internship. Although previous studies have examined video-based learning and simulation independently, less attention has been paid to how these resources are pedagogically integrated within time-constrained clinical environments and how such integration shapes students’ engagement with procedural learning.

## Methods

### Study Design

This study used an interpretive qualitative design to explore how medical students and an instructor experienced the integration of asynchronous instructional videos and simulation-based practice within a surgical internship. A qualitative approach was appropriate because the aim was not to measure technical performance but to examine participants’ perceptions and experiences regarding these educational strategies in a real-world clinical training context. This design enabled an in-depth understanding of how learners engaged with preparatory video resources and how these were integrated with hands-on simulation during a clinically demanding stage of training.

Data were analyzed using reflexive thematic analysis, following the approach described by Braun and Clarke [16,17]. This approach supported the identification of patterns of meaning across participants’ accounts. Consistent with its epistemological assumptions, the analytic process emphasized reflexive interpretation rather than coding reliability or consensus.

### Description of the Intervention

The intervention consisted of a suturing simulation module supported by asynchronous instructional videos implemented at the beginning of the surgical internship.

The asynchronous component included 5 short instructional videos (approximately 5 minutes each) demonstrating simple interrupted, continuous, vertical mattress, horizontal mattress, and intradermal sutures. Viewing was recommended but not mandatory, and engagement was not formally monitored.

The in-person component consisted of a supervised 2-hour workshop using individual simulation models and suturing kits. The instructor provided real-time feedback, and the videos remained accessible during practice.

### Participants and Recruitment

Participants were sixth-year medical students enrolled in a surgical internship. All students who completed the module were eligible.

Approximately 1 month after the intervention, students were invited in person by a member of the research team. Participation was voluntary and unrelated to academic evaluation.

A total of 20 students participated. They were typically aged 23 to 25 years and had prior exposure to suturing with variable practice opportunities.

One faculty instructor also took part in a semistructured interview. The instructor had experience teaching procedural skills and did not participate in student evaluation.

Sample adequacy was considered appropriate based on the concept of information power, which suggests that studies addressing focused research aims with specific and information-rich samples may require fewer participants to generate meaningful qualitative insights. In this study, the aim was narrowly defined, and participants shared a highly specific educational context.

In addition, the use of focus groups and an instructor interview enabled the generation of rich, experience-based data. The application of reflexive thematic analysis supported an in-depth interpretation of participants’ accounts, contributing to sufficient information power to address the study aim.

### Data Collection

Data were collected through focus groups with students and a semistructured interview with the instructor.

Two focus groups were conducted, each including approximately 8 to 12 students, for a total of 20 participants. Each session lasted approximately 75 minutes and was conducted in person in a meeting room within the medical school. Focus groups took place approximately 1 month after completion of the suturing simulation module, allowing participants to reflect on their experiences after initial exposure to clinical practice during the surgical internship.

In addition, a semistructured individual interview lasting approximately 45 minutes was conducted with the instructor who facilitated the workshop. The interview explored the instructor’s perspectives on the design, implementation, and perceived value of the intervention.

A semistructured discussion guide was developed by the research team based on the study objectives and informed by existing literature on simulation-based and video-assisted learning in medical education. The guide was designed to explore key experiential domains aligned with the research aim, including participants’ perceptions of the asynchronous videos, experiences during the simulation workshop, perceived facilitators and barriers to learning, and perceived relevance to clinical practice.

These domains were defined to reflect the study’s interpretive focus on how participants experienced and made sense of the integration of digital and simulation-based learning strategies within a clinical training context.

The guide was developed collaboratively by the research team and refined through iterative discussion to ensure clarity, relevance, and alignment with the study objectives prior to data collection.

All focus groups and the interview were audio-recorded with participants’ consent, transcribed verbatim, and pseudonymized prior to analysis.

Focus groups were moderated by a member of the research team who was not involved in student assessment or direct clinical supervision to reduce potential hierarchical influences on participants’ responses.

### Data Analysis

Data were analyzed using reflexive thematic analysis, following the approach described by Braun and Clarke [14]. The analysis involved an iterative and recursive process, including familiarization with the data, generation of initial codes, development of candidate themes, review and refinement of themes, and production of the final analytic narrative.

All transcripts were read multiple times to achieve immersion in the data. Initial coding was conducted in a data-driven manner, allowing codes to be generated inductively from participants’ accounts. Four members of the research team participated in the analytic process. Coding and theme development were conducted through ongoing, collaborative engagement with the data, supported by regular analytic meetings in which interpretations were discussed, codes refined, and themes developed.

Consistent with the principles of reflexive thematic analysis, these discussions were not intended to establish interrater reliability or achieve coding consensus but rather to support reflexive engagement with the data and deepen interpretive analysis.

During the later stages of analysis, the Technological Pedagogical Content Knowledge (TPACK) framework was used as a sensitizing lens to interpret how technological resources (asynchronous videos), pedagogical strategies (simulation-based instruction), and disciplinary content (suturing techniques) interacted within participants’ experiences [18]. This interpretive lens supported the organization and contextualization of themes without constraining the inductive coding process.

In addition, analytic attention was given to cross-cutting experiential dimensions, including motivation, perceived learning, facilitators, barriers, and perceived transfer to clinical practice, which were used as organizing categories to support thematic interpretation. This study was reported in accordance with the Consolidated Criteria for Reporting Qualitative Research (COREQ) guidelines. A complete checklist is provided as Checklist 1.

The research team included faculty members and medical students with experience in medical education. Throughout the analytic process, the research team acknowledged their dual role as educators and researchers and engaged in reflexive discussions to consider how this positioning might influence data interpretation.

No qualitative data analysis software was used. Data organization and coding were conducted manually by the research team through iterative and collaborative engagement with the transcripts.

### Ethical Considerations

The study was approved by the scientific ethics committee of *Universidad San Sebastián* (40-25). Ethics approval was obtained prior to the commencement of data collection. All participants received information about the study and provided informed consent prior to participation. Participation was voluntary, and students were explicitly informed that their decision to participate or decline would not affect their academic evaluation or standing. All data were anonymized and managed confidentially throughout the research process. Participants did not receive financial or other compensation for their participation.

## Results

### Participants Characteristics

The study included 20 sixth-year medical students who were beginning their surgical internship and had completed a simulated suturing module supported by asynchronous instructional videos, as well as 1 faculty member who facilitated the workshop and supervised students during their clinical training. Students were typically aged between 23 and 25 years and had prior introductory exposure to suturing techniques, although opportunities for repeated supervised practice varied.

All participating students had completed the full educational module prior to taking part in the focus groups. All participants completed the data collection activities, and no withdrawals were recorded.

### Primary Results

Analysis of the focus groups and the faculty interview revealed convergent perceptions regarding the use of asynchronous videos in combination with hands-on practice using simulation models during the surgical internship. Participants described how this combined approach was experienced and used within a context characterized by high clinical demands and limited time for structured teaching.

The findings are organized into four interrelated themes: (1) preparation for hands-on practice, (2) integration of asynchronous videos and simulation-based learning, (3) perceived confidence and readiness for clinical tasks, and (4) logistical and contextual constraints.

### Preparation for Hands-on Practice

Students described asynchronous videos as a preparatory resource that supported their engagement with hands-on practice, allowing them to approach the workshop with prior familiarity with the procedures and reducing the need for real-time procedural recall. They emphasized the ability to access the content at their own pace, review specific segments, and engage with the material without interfering with clinical responsibilities:

The videos were short, well summarized, and concise, so they didn’t take much time to review.

[Student, focus group 1]

Several participants highlighted that this flexibility was particularly relevant within the demanding schedule of the surgical internship:

Not everyone has the opportunity to practice at home, so having this instance is really helpful, especially at the beginning of the internship.

[Student, focus group 2]

### Integration of Asynchronous Videos and Simulation-Based Practice

Participants described the combination of prior video exposure and supervised hands-on practice as enabling more focused engagement during the workshop, shifting attention from procedural recall to technical execution. Students reported that reviewing the procedures beforehand allowed them to focus during the workshop on performing the techniques rather than recalling procedural steps:

The tutor played the videos while we were doing the activity, which really helped guide us during practice.

[Student, focus group 1]

Being able to see the procedures beforehand allowed me to focus more on doing the sutures properly instead of thinking about what the next step was.

[Student, focus group 1]

Students also described the availability of individual simulation kits as a facilitator of continuous practice during the session:

It was great that each student had their own kit, because we didn’t have to wait for others to practice.

[Student, focus group 2]

### Perceived Confidence and Readiness for Clinical Tasks

Students described a sense of increased confidence and perceived readiness when approaching early clinical tasks, which they associated with having had the opportunity to engage in structured and supported practice prior to clinical exposure:

It helped me start clinical practice feeling more confident, because suturing is something that’s requested from the very first shifts.

[Student, focus group 2]

Some participants also noted that the workshop provided an opportunity to revisit and practice skills that were often assumed to be already acquired but not consistently reinforced under supervision:

It’s something that people often assume you already know how to do well, but in reality you only learn by practicing.

[Student, focus group 1]

### Logistical and Contextual Constraints

Although participants generally described the module positively, they also identified challenges related to time constraints and limited opportunities for individualized feedback during the workshop:

There were some sutures where I wasn’t sure if they were right or wrong, because there wasn’t enough time to review everyone’s work.

[Student, focus group 2]

These constraints were described in relation to the number of students per instructor and the limited duration of the session.

From the faculty perspective, the use of asynchronous videos was described as supporting a more focused use of in-person time, allowing attention to be directed toward technical aspects and student questions:

They arrived with a prior foundation, which made it possible to focus more on improving technique rather than explaining everything from scratch.

[Faculty member]

The faculty member also identified logistical challenges related to the physical space and audio conditions, while noting that prior exposure to the videos influenced how the workshop was experienced:

The difficulties were more related to the physical space than to the videos themselves; the important thing was that they had already reviewed the material beforehand.

[Faculty member]

### Perceived Applicability to Other Areas of Training

Participants described the potential applicability of this educational approach to other areas of the surgical internship, particularly for skills that are expected in clinical practice but not always explicitly taught:

It would be useful to have this kind of preparation in other areas as well, like trauma or wound care, because these are skills that are expected but not always taught beforehand.

[Student, focus group 2]

From the faculty perspective, the experience was described as a potential basis for broader integration of audiovisual resources and simulation within internship training:

It’s a tool that could be maintained and used with other groups of students.

[Faculty member]

## Discussion

### Principal Findings

This study explored how medical students and an instructor experienced an asynchronous video-supported suturing simulation module during a surgical internship. Overall, participants described the integrated use of asynchronous videos and supervised simulation-based practice as a useful and contextually relevant educational strategy in a setting characterized by high clinical demands and limited protected teaching time.

Across themes, participants highlighted the role of asynchronous videos as a preparatory resource, the integration of video-based and hands-on learning during the workshop, and a perceived sense of confidence and readiness when approaching early clinical tasks. At the same time, logistical constraints related to time, feedback opportunities, and physical space were also identified.

### Interpretation in Relation to Existing Literature

The findings of this study align with prior research suggesting that asynchronous video-based learning may support flexible access to instructional content and allow learners to engage with procedural knowledge at their own pace. In this study, participants described how this flexibility was particularly relevant within the demanding context of a surgical internship, where time for structured learning is often limited [4,5]. Recent studies have further emphasized the role of asynchronous video-based learning in supporting self-regulated learning and flexible engagement in clinically intensive training environments, reinforcing its relevance in contexts with competing clinical demands [19,20].

In addition, the perceived value of combining asynchronous videos with simulation-based practice is consistent with the literature emphasizing the role of multimodal educational strategies in procedural skills training. Rather than functioning as a replacement for supervised instruction, participants described asynchronous videos as a preparatory scaffold that enabled more focused engagement during hands-on practice [7,10]. More recent literature has highlighted that integrating digital resources with simulation-based instruction may enhance the efficiency of in-person training by allowing learners to arrive with foundational procedural understanding, thereby optimizing opportunities for feedback and technical refinement [21,22].

Importantly, this study extends the existing literature by providing qualitative insights into how this combination of resources is experienced within a real-world clinical training environment [23]. Although previous studies have examined video-based learning or simulation independently, the findings of this study highlight how their integration may be perceived as supporting the organization and use of limited instructional time during surgical internships [24]. In this sense, this study contributes to a growing body of research that moves beyond evaluating isolated educational tools and instead examines how integrated pedagogical designs are experienced and enacted within authentic clinical training settings.

A key contribution of this study lies in conceptualizing asynchronous videos not merely as supplementary resources but as a form of preparatory pedagogical scaffolding within clinically demanding training environments. This scaffolding appears to enable a redistribution of cognitive and instructional effort, allowing supervised sessions to focus more explicitly on technical refinement and feedback. In this sense, the integration of digital and simulation-based resources may be understood as a strategy for optimizing the pedagogical use of limited clinical teaching time.

The use of the TPACK framework as an interpretive lens further supports understanding how technological resources (videos), pedagogical approaches (simulation-based instruction), and disciplinary content (suturing techniques) interact within situated learning experiences. From a TPACK perspective, participants’ experiences suggest that the perceived value of the intervention emerged from the interaction among technological resources (videos), pedagogical design (simulation-based instruction), and disciplinary content (suturing skills), rather than from any single component in isolation [8].

From a curricular perspective, these findings suggest that asynchronous instructional videos may function as preparatory scaffolding that allows simulation-based workshops to focus more effectively on hands-on practice and feedback. In contexts where teaching time is constrained, such as surgical internships, this approach may enable a more pedagogically efficient use of in-person instructional time [7,11].

Participants’ accounts also highlight the importance of ensuring adequate opportunities for individualized feedback and appropriate instructor-to-student ratios during simulation sessions. Additionally, logistical factors such as physical space and audio conditions were described as influencing the learning experience, suggesting that implementation requires attention not only to educational design but also to practical conditions.

The perceived applicability of this approach to other areas of clinical training suggests potential for broader integration of asynchronous and simulation-based strategies within undergraduate medical education. However, such adaptations should consider contextual factors and resource availability.

### Limitations

Several limitations should be considered when interpreting these findings. First, the study was conducted within a single medical school and involved students from one internship cohort, which may limit the transferability of the results to other institutional contexts. Second, the faculty perspective was represented by a single instructor interview, which restricts the diversity of teaching perspectives included in the analysis.

Third, the study focused on participants’ perceptions and did not include objective measures of procedural skill performance. In addition, data collection was conducted approximately one month after completion of the intervention. This timing may have influenced participants’ recollections, as their experiences could have been shaped by subsequent clinical exposure during the surgical internship. This temporal distance may have introduced recall bias and influenced how participants interpreted and evaluated the perceived usefulness of the module. Social desirability bias during focus group discussions may also have shaped participants’ responses.

Engagement with the asynchronous video component was not formally measured, and individual exposure to the videos may have varied across participants. Educational practices and clinical training structures in Chile may also differ from those in other regions, which should be considered when interpreting the broader applicability of the findings.

The findings are best understood as contextually situated insights that may inform transferability to similar educational settings rather than broad generalization.

Finally, the involvement of the research team in the educational context may have influenced participants’ responses, which was addressed through reflexive discussions during the analytic process.

### Conclusions

This study provides qualitative insights into how medical students and an instructor experienced the integration of asynchronous video resources and simulation-based practice within a surgical internship. Participants perceived this combined approach as supporting preparation for hands-on learning and shaping how supervised training time was used. These findings suggest that asynchronous instructional videos may function as a form of preparatory pedagogical scaffolding that enables a more focused and efficient use of simulation-based training within clinically demanding environments. Further research is needed to explore how such approaches can be adapted across different contexts and to examine their relationship with objective measures of procedural learning.

## Supplementary material

10.2196/90563 Checklist 1 COREQ checklist.

## Abbreviations

COREQ: Consolidated Criteria for Reporting Qualitative Research
TPACK: Technological Pedagogical Content Knowledge
