# Supplementary material for: Medical Students’ Experiences With an Integrated Surgical Suturing Training Module Using Simulation Models and Asynchronous Videos: Interpretive Qualitative Study
Source: JMIR Med Educ. 2026 Jul 9;12:e90563. doi: 10.2196/90563 (PMC13348991; doi:10.2196/90563)
Supplement: Checklist 1 [file mededu-v12-e90563-s001.pdf]

COREQ (Consolidated criteria for Reporting Qualitative research) Checklist.

Domain 1: Research Team and Reflexivity

**No.**	**Item**	**Guide Question / Description**	**Reported**	**Location in Manuscript**
1	Interviewer/facilitator	Which author conducted the focus groups/interview?	Yes	Methods – Data Collection
2	Credentials	What were the researcher's credentials?	Yes	Methods – Research Team
3	Occupation	What was their occupation at the time?	Yes	Methods – Research Team
4	Gender	Was the researcher's gender reported?	No	Not reported
5	Experience and training	What experience/training did the researcher have?	Yes	Methods – Research Team
6	Relationship established	Was a relationship established prior to study?	Yes	Methods – Data Collection
7	Participant knowledge of interviewer	What did participants know about researcher?	Yes	Methods – Recruitment
8	Interviewer characteristics	Any biases, assumptions reported?	Yes	Methods – Reflexivity

Domain 2: Study Design

No.	Item	Guide Question / Description	Reported	Location in Manuscript
9	Methodological orientation	What methodological approach was used?	Yes	Methods – Study Design
10	Sampling	How were participants selected?	Yes	Methods – Participants
11	Method of approach	How were participants approached?	Yes	Methods – Recruitment
12	Sample size	How many participants?	Yes	Methods – Participants
13	Non-participation	Refusals or dropouts?	Yes	Results – Participant Characteristics
14	Setting of data collection	Where was data collected?	Yes	Methods – Data Collection
15	Presence of non-participants	Anyone else present?	No	Not reported
16	Description of sample	Key characteristics of participants	Yes	Results – Participant Characteristics

Domain 3: Data Collection

No.	Item	Guide Question / Description	Reported	Location in Manuscript
17	Interview guide	Were questions provided?	Yes	Methods – Data Collection
18	Repeat interviews	Were repeat interviews done?	No	Not reported
19	Audio/visual recording	Were sessions recorded?	Yes	Methods – Data Collection
20	Field notes	Were field notes made?	No	Not reported
21	Duration	Duration of interviews/FGs	Yes	Methods – Data Collection
22	Data saturation	Was saturation discussed?	No (information power used)	Methods – Participants
23	Transcripts returned	Were transcripts returned to participants?	No	Not reported

Domain 4: Data Analysis

No.	Item	Guide Question / Description	Reported	Location in Manuscript
24	Number of data coders	How many coded data?	Yes (4 researchers)	Methods – Data Analysis
25	Description of coding tree	Coding structure described?	Yes	Methods – Data Analysis
26	Derivation of themes	Inductive or deductive?	Yes	Methods – Data Analysis
27	Software	Software used?	No	Not reported
28	Participant checking	Feedback on findings?	No	Not reported

Domain 5: Reporting

No.	Item	Guide Question / Description	Reported	Location in Manuscript
29	Quotations presented	Participant quotes used?	Yes	Results
30	Data and findings consistent	Alignment between data and findings	Yes	Results
31	Clarity of major themes	Clearly presented?	Yes	Results
32	Clarity of minor themes	Minor themes described?	Yes	Results
33	Negative cases described	Contradictions included?	Yes	Results – Theme 4
